# C_4_ photosynthetic machinery: insights from maize chloroplast proteomics

**DOI:** 10.3389/fpls.2013.00085

**Published:** 2013-04-15

**Authors:** Qi Zhao, Sixue Chen, Shaojun Dai

**Affiliations:** ^1^Key Laboratory of Saline-alkali Vegetation Ecology Restoration in Oil Field, Ministry of Education, Alkali Soil Natural Environmental Science Center, Northeast Forestry UniversityHarbin, China; ^2^Department of Biology, Genetics Institute, Interdisciplinary Center for Biotechnology Research, University of FloridaGainesville, FL, USA

**Keywords:** maize, chloroplast, proteomics, C_4_ plant, photosynthesis

## Abstract

C_4_ plants exhibit much higher CO_2_ assimilation rates than C{}_3_ plants under certain conditions. The specialized differentiation of mesophyll cell and bundle sheath cell type chloroplasts is unique to C_4_ plants and improves photosynthetic efficiency. Maize (*Zea mays*) is an important crop and model with C_4_ photosynthetic machinery. 2DE and high-throughput quantitative proteomics approaches (e.g., isobaric tags for relative and absolute quantitation and shotgun proteomics) have been employed to investigate maize chloroplast structure and function. These proteomics studies have provided valuable information on C_4_ chloroplast protein components, photosynthesis, and other metabolic mechanisms underlying chloroplast biogenesis, stromal, and membrane differentiation, as well as response to salinity, high/low temperature, and light stress. This review presents an overview of proteomics advances in maize chloroplast biology.

## INTRODUCTION

Chloroplasts are organelles for photosynthesis. Chloroplasts also participate in the amino acid, vitamin, isoprenoid, and lipid biosynthesis, as well as reduction of nitrite and sulfate (van Wijk, 2000; Baginsky and Gruissem, 2004). A previous study has proposed that there are ~3000 proteins in mature chloroplasts that have specialized distributions and functions (Leister, 2003). Based on the primary product of carbon fixation, plants are classified as C_3_ and C_4_ species. Oxaloacetate (a four-carbon compound) and 3-phosphoglycerate (a three-carbon compound) are the primary products of carbon assimilation in the C_4_ and C_3_ plants, respectively. Under certain conditions, the CO_2_ assimilation rate of C_4_ plants is much higher than that of C_3_ plants. In addition, C_4_ photosynthesis enables higher nitrogen and water use efficiency than C_3_ photosynthesis. Maize (*Zea* *mays*) is a representative C_4_ plant of the nicotinamide adenine dinucleotide phosphate (NADP)-malic enzyme type. Primary carbon fixation and reduction are spatially separated between two different cell types, mesophyll cells (M) and bundle sheath cells (BS). C_4_ plant M and BS have morphologically and biochemically distinct features and cooperate in photosynthesis (Edwards et al., 2001; Majeran et al., 2005). C_4_ chloroplasts in M contain grana thylakoids, linear electron transport, and product reduced NADP (NADPH); while chloroplasts in BS are agranal and depleted of photosystem II (PSII), and perform most of the Calvin cycle reactions. The differentiation of C_4_ chloroplasts in different cell types is regulated by a complex network at both gene (Sawers et al., 2007) and protein (Majeran et al., 2005) levels. The C_4_ photosynthetic mechanism is a sophisticated signaling and metabolic network. In the past 7–8 years, high-throughput quantitative proteomics studies on the C_4_ plant maize chloroplasts have been carried out. These proteomics investigations, mainly carried out by the van Wijk’s lab, have been able to consolidate previous scattered information and provide new insights into the fine-tuned chloroplast biogenesis/differentiation in the M and BS, chloroplast stress response, and toward understanding C_4_ photosynthetic machinery. Especially, the studies from van Wijk’s lab provided several new insights into NADPH type C_4_ photosynthesis and the distribution of protein functions across BS and M chloroplasts (Majeran et al., 2005, 2008; Covshoff et al., 2008; Majeran and van Wijk, 2009; Friso et al., 2010). In this mini review, we mainly aim to present a brief summary of current quantitative proteomics studies of maize chloroplasts.

## C_4_ CHLOROPLAST STROMAL PROTEOME IN M AND BS

Chloroplast stromal proteins from maize M and BS were identified using gel-based and gel-free proteomics approaches (Majeran et al., 2005; Friso et al., 2010). The differentially accumulated proteins from the two types of chloroplasts are mainly involved in primary metabolisms, redox regulation, gene expression, and protein homeostasis. The proteomics results yield several new insights into cellular specialization of the C_4_ photosynthesis.

**FIGURE 1:**
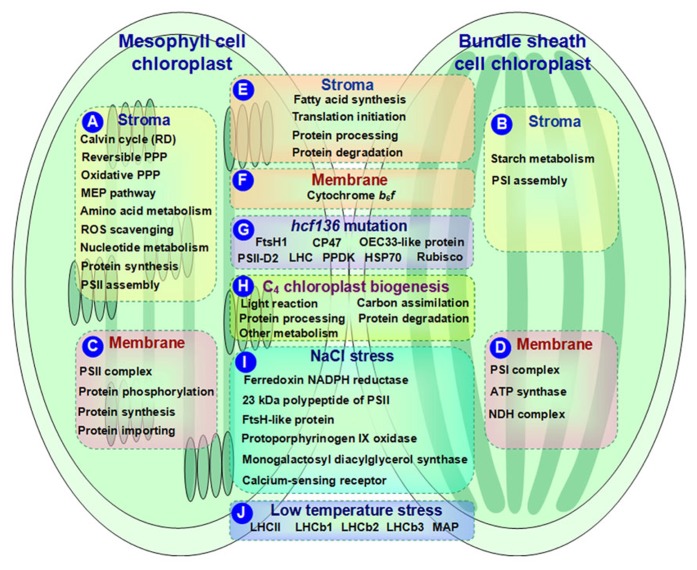
**Schematic presentation of cell-specific or stress-responsive pathways and proteins in maize chloroplasts revealed from proteomics studies.** **(A,B)** Preferential metabolic pathways in the stroma of M and BS, respectively; **(C,D)** Preferential metabolic pathways and protein complexes in the chloroplast membrane of M and BS, respectively; **(E,F)** Metabolic pathways and protein complexes equally distributed in the chloroplast stroma/membrane of M and BS; **(G)** Differentially expressed proteins in chloroplasts of maize *hcf136* mutant in comparison to wild-type; **(H)** Dynamics of metabolic pathways during maize chloroplast biogenesis; **(I)** Salt-responsive proteins in maize chloroplasts; **(J)** Low temperature-responsive proteins in maize chloroplasts. ATP, adenosine triphosphate; HSP, heat shock protein; LHC, light harvesting complex; MAP, minor antenna proteins; MEP, methylerythritol phosphate; NADPH, reduced nicotinamide adenine dinucleotide phosphate; NDH, NAD(P)H dehydrogenase; OEC, oxygen evolving center; PPDK, pyruvate orthophosphate dikinase; PPP, pentose phosphate pathway; PSI, photosystem I; PSII, photosystem II; RD, reductive phase; ROS, reactive oxygen species; Rubisco, ribulose-1,5-bisphosphate carboxylase/oxygenase.

Cell type-specific distribution of many biochemical processes exist in the M and BS chloroplasts (**Figures 1A, B**). For instance, the reductive phase of the Calvin cycle, reversible pentose phosphate pathway (PPP), oxidative PPP, methylerythritol phosphate pathway, and amino acid metabolism (e.g., biosynthesis of arginine, branched amino acids, and aromatic amino acids) are more active in the M chloroplasts than that in BS (Majeran et al., 2005; Friso et al., 2010). Based on proteomics results, most of the enzymes involved in the above processes were preferentially expressed in the M chloroplasts, suggesting high demand for various metabolites in the M chloroplasts (Friso et al., 2010; **Figure 1A**). In contrast, most of the enzymes involved in starch metabolism were more abundant in the BS chloroplasts than in the M chloroplasts (**Figure 1B**). This is consistent with the fact that BS chloroplasts possess more starch particles. However, various proteins involved in fatty acid synthesis were equally distributed across M and BS chloroplasts (**Figure 1E**), indicating the similar demands for fatty acids in both M and BS chloroplasts (Majeran et al., 2005; Friso et al., 2010).

Reactive oxygen species (ROS) production and redox balance play important roles in regulating plastid functions (Baier and Dietz, 2005). Proteomics results revealed that the majority of ROS scavenging enzymes showed high abundance in the M chloroplasts (**Figure 1A**). This is proposed to be associated with high linear electron transport rate and water-splitting activity of PSII in the M chloroplasts (Majeran et al., 2005; Majeran and van Wijk, 2009). In addition, a great portion of nucleotide metabolism-related enzymes, such as adenylate monophosphate kinase 2, nucleoside diphosphate kinase 2, soluble inorganic pyrophosphatase, and membrane-bound adenosine triphosphate (ATP)/adenosine diphosphate (ADP) translocator, showed preferential accumulation in the M chloroplasts (Friso et al., 2010; **Figure 1A**). Since *de novo* biosynthesis of nucleotides is energy consuming, the M chloroplasts could generate adequate energy through linear and cyclic electron transport (Zrenner et al., 2006).

The components of M and BS chloroplast protein synthesis machineries show overlapping but different expression patterns in C_4_ plants. Comparative proteomics analysis showed that the majority of initiation and elongation factors (involved in protein translation initiation), general chaperones (related to protein processing), and Clp proteases (participate in protein degradation) were equally distributed across M and BS chloroplasts (**Figure 1E**). In contrast, ribosomal proteins and tRNA synthases, involved in protein synthesis, were much higher in the M chloroplasts than in the BS (**Figure 1A**). This implies that in the M chloroplasts, there is more protein synthesis which is required for repairing the chloroplast-encoded reaction center protein D1 (Baena-Gonzalez and Aro, 2002). Additionally, assembly factors for PSII complexes showed higher abundance in the M chloroplasts (**Figure 1A**), while photosystem I (PSI) complex assembly factors were preferentially expressed in the BS chloroplasts (**Figure 1B**). The well-correlated expression of proteins in the M and BS chloroplasts suggests existence of well-developed regulatory networks in C_4_ photosynthesis (Friso et al., 2010).

## C_4_ CHLOROPLAST MEMBRANE PROTEOME IN M AND BS

Maize thylakoid membrane proteins play key roles in C_4_ photosynthesis. Thirty-four thylakoid membrane proteins were identified and quantified using shotgun proteomics approaches (Liu et al., 2011). The majority of the proteins (~76%) were involved in photosynthetic light reactions. Among them, only two PSI subunits were detected, suggesting that most of the PSI components accumulated at lower levels. In addition, a comparative proteomics study on the M chloroplast envelopes between maize and C_3_ plant pea (*Pisum sativum*) revealed that C_4_- and C_3_-type chloroplasts contained qualitatively similar but quantitatively different membrane protein components (Brautigam et al., 2008). For instance, several translocators (e.g., outer envelope porin, triosephosphate translocator, and phospho*enol*pyruvate translocator) showed higher abundance in C_4_ chloroplast envelopes than in C_3_ plants. However, two protein import complex components, Tic55 and ClpC/Hsp93, were found at lower expressional levels in C_4_ chloroplast envelopes (Brautigam et al., 2008). These data imply that the C_4_ chloroplast envelope transporters are adapted to meet the demand of high metabolic flux rates during C_4_ photosynthesis. However, the small number of proteins identified in these studies provides limited information toward understanding the dynamics and functions of C_4_ thylakoid membrane proteins.

Current quantitative proteomics approaches (e.g., isobaric tags for relative and absolute quantitation (iTRAQ) and label-free quantification) provide more information for understanding the differentiation and oligomeric states of membrane proteins in the C_4_ chloroplasts of BS and M (Majeran et al., 2008; Friso et al., 2010). For instance, the contents of PSI and PSII complexes were more abundant in the BS (**Figure 1D**) and M (**Figure 1C**) thylakoids, respectively. This is consistent with their specific roles in the corresponding cell types. Besides, ATP synthase was increased in the BS thylakoids (**Figure 1D**), while cytochrome *b*_6_f was unchanged between the two cell types (**Figure 1F**). In addition, NAD(P)H dehydrogenase (NDH) complex showed a preferential BS accumulation (Ivanov et al., 2007; **Figure 1D**). NDH is involved in chlororespiration and cyclic electron flow around PSI. A novel subcomplex of NDH complex was also identified in the BS thylakoids (Majeran et al., 2008), which is speculated to be responsible for carbon concentrating especially when ribulose-1,5-bisphosphate carboxylase/oxygenase (Rubisco) carboxylation rate is lower than the malic enzyme decarboxylation rate.

Proteomics analysis revealed that different paralogs with cell-specific accumulation patterns existed in the M and BS chloroplast membranes. For example, two light harvesting complex II (LHCII) members exhibited higher BS/M ratios than other LHCII proteins (Majeran et al., 2008). Additionally, the differential BS/M ratios of PSI subunits paralogs might reflect the distinct PSI composition between the BS and M thylakoids (Majeran et al., 2008). Further studies assisted by high quality annotated maize genome sequences are needed for exploring the subtle but interesting differences.

The ROS scavenging system and (de)phosphorylation-driven protein state transitions have been employed by C_4_ plants during the M and BS differentiation to avoid light stress and optimize light harvesting capacity. Proteomics results showed that the low BS/M ratio for lumenal peroxidase-Q but high BS/M ratio for peroxidase-E implied the distinct ROS scavenging mechanisms in the M and BS chloroplasts. Several light stress protein homologs exhibited preferential accumulation in the M chloroplasts (Majeran et al., 2008). Furthermore, thylakoid kinases, such as state transition kinase (STN) 7, STN8, phosphoprotein TSP9, and lumenal isomerase TLP40, exhibited low BS/M ratios (Majeran et al., 2008; **Figure 1C**). These enzymes may be involved in (de)phosphorylation-driven protein state transitions in the M thylakoids to balance the excitation of PSI and PSII reaction centers.

To maintain protein homeostasis within different cell types, C_4_ plants differentiated distinct machineries for protein synthesis, assembling, importing, processing, and degradation in the M and BS chloroplasts. The differentially accumulated proteins in maize chloroplast membranes of the M and BS support the following metabolic mechanisms: (1) The preferential M accumulation of ribosomal proteins suggest high translation rates in the M chloroplasts, which can contribute to the high abundance of PSII subunits and short lifetime of PSII reaction center proteins caused by light-induced damage (Friso et al., 2010; **Figure 1C**). (2) In terms of protein assembly, the preferential accumulation of low PSII accumulation 1 protein (LPA1) in the M chloroplasts is consistent with its role in D1 protein synthesis (Peng et al., 2006). However, it was intriguing that high chlorophyll fluorescence 136 (HCF136) responsible for assembling PSII reaction centers (Covshoff et al., 2008) was equally distributed in the BS and M chloroplast membranes. Its specific functions in the two cell types remains to be determined (Majeran et al., 2008). (3) Highly accumulated protein importing-related proteins (e.g., Tic110, Tic21, and Tic40) in the M chloroplasts indicate an increased protein flux in the M chloroplasts (Majeran et al., 2008; **Figure 1C**). (4) As for protein degradation, lumenal DegP1 was enriched in the M thylakoids and this is consistent with its function in D1 protein degradation (Sun et al., 2007). Several proteolysis-related proteins showed distinct BS/M ratios, indicating their specific roles in the BS and M chloroplasts (Majeran et al., 2008). The role of proteolysis in the BS and M differentiation needs to be further explored by studying the cell type-specific accumulation of proteases during BS/M development.

Key gene mutations of the M and BS chloroplasts in C_4_ plants provide valuable information toward understanding the mechanisms underlying the C_4_ photosynthetic machinery. Covshoff et al. (2008) identified an *Activator*-induced maize mutant that lacks PSII activity. This mutated gene is a homolog of *HCF136*, which is responsible for PSII assembly or stability (Plucken et al., 2002). *hcf136* mutant seedlings contained smaller chloroplasts in both M and BS and abnormal/no grana in the M plastids (Covshoff et al., 2008). Furthermore, PSII reaction center functionality was undetected (*F*_v_/*F*_m_ = 0) in mutant plants. Consistently, PSII reaction center and core subunits were absent from the *hcf136* thylakoid membranes, while PSI was not affected (Covshoff et al., 2008). Moreover, major LHCII in the mutant seedlings displayed a monomeric form instead of the typical trimeric form of wild-type thylakoids (Covshoff et al., 2008). Additionally, the *psbB-psbH-psbT-petB-petD* polycistron, encoding the components of PSII (e.g., *psbB*, *psbH*, *psbN*, and *psbT*) and cytochrome *b*_6_f (e.g., *petB* and *petD*), was misprocessed in the *hcf136 *mutant M (Covshoff et al., 2008). These results prove that the mutation of *hcf136* leads to disruption of PSII assembly or stability. Proteomics analysis found that *hcf136* mutation led to differential accumulation of several proteins in the thylakoid membranes. These proteins were identified as FtsH1, CP47, oxygen evolving center 33-like protein, PSII-D2, LHC, pyruvate orthophosphate dikinase, heat shock protein 70 (HSP70), and Rubisco small subunit (Covshoff et al., 2008; **Figure 1G**). However, the relative levels of the gene transcripts did not correlate with corresponding protein levels. This inconsistency between transcript accumulation and protein abundance suggests the involvement of transcriptional/translational regulations during C_4_ differentiation.

## CHANGES IN PROTEIN ABUNDANCE DURING C_4_ CHLOROPLAST BIOGENESIS

Maize greening is accompanied by the differentiation of the M and BS chloroplasts for C_4_ photosynthesis. This process has long been considered as a model system to study the sophisticated mechanisms of chloroplast biosynthesis. A large-scale proteomics analysis of the leaf and the BSs with their vascular bundle along the leaf developmental gradient has provided detailed dynamic information of more than 4300 proteins for a systems-level understanding of maize leaf formation and differentiation (Majeran et al., 2010). The changes of protein expression patterns highlighted the active transition and/or differentiation of C_4_ malate-pyruvate shuttle, photosynthetic linear and cyclic electron flow, photorespiration, protein translation, specific transporters, and other metabolic processes along the leaf developmental gradient (Majeran et al., 2010). Hierarchical clustering of protein expression data revealed obvious spatial differentiation characteristics. The chloroplast biogenesis-related proteins accumulated to significant levels in the first 4 cm from the leaf base, and the majority of the photosynthetic apparatus-related proteins started to accumulate significantly beyond the 4 cm from ligule. This indicates that the establishment of basic chloroplast functions takes place prior to the specific cell-type differentiation related to C_4_ functions (Majeran et al., 2010).

In addition to leaf proteomics, a chloroplast proteomics study also revealed protein changes during maize greening (0~48 h) (Lonosky et al., 2004; **Figure 1H**). (1) Proteins involved in light reactions changed during greening. For instance, ATPase is the most abundant protein identified in maize chloroplasts during greening. In general, the four subunits of ATPase increased continually with greening (0 ~48 h). However, some α and β subunits showed different expression patterns possibly due to the demands for different protein forms during specific chloroplast differentiation periods. (2) In general, photosynthetic carbon assimilation-related enzymes were increased during the early time (0~4 h) of greening. This is consistent with a previous notion that the plastid assembles the photosynthetic apparatus during early development. Afterward, some enzymes such as β-amylase, NADP malate dehydrogenase, and phosphoglycerate kinase (PGK) reached a plateau. Interestingly, two enzymes displayed opposite expression patterns. The levels of glyceraldehyde-3-phosphate dehydrogenase (GAPDH) kept increasing, while isoamylase began to decrease after the early phase. Moreover, the expression patterns of PGK and GAPDH were consistent with their mRNA levels reported in previous studies (Dewdney et al., 1993; Bringloe et al., 1996). (3) The plastid chaperonins and proteases also changed during maize greening. They are involved in protein processing and degradation, respectively. For instance, α subunit of the 60-kD and 20-kD chaperonins displayed moderate increases in the early (0~4 h) and middle (12 h) phases but decreased at 48 h. In addition, both HSP70 and ClpC increased during the initial phase of greening and reached a plateau at 48 h. These data imply that the active protein folding and degradation takes place, which contribute to active alterations of protein activity and turnover in various signaling and metabolic changes during chloroplast biogenesis. (4) Proteins involved in various plastid metabolic processes (e.g., acetyl-coA carboxylase, beta-D-glucosidase, nucleic acid-binding protein) showed complex patterns of protein abundances during greening. All the above findings have provided valuable insights into the mechanisms underlying chloroplast biogenesis.

## STRESS-RESPONSIVE PROTEINS IN C_4_ CHLOROPLASTS

Salinity is thought to have a strong influence on plant chloroplast protein composition. Several salt-responsive proteins have been identified in maize chloroplasts undergoing 25 mM NaCl treatment for 4 h using 2DE-based proteomics approaches (Zorb et al., 2009; **Figure 1I**). In the salt-stressed maize plants, three photosynthesis-related proteins (i.e., ferredoxin NADPH reductase, 23 kDa polypeptide of PSII, and FtsH-like protein) were increased under NaCl stress. This would help to attenuate the severe effects of Na^+^ on the photosynthetic machinery. Additionally, the enhanced abundance of protoporphyrinogen IX oxidase was detected in salt-treated maize chloroplasts. This enzyme is involved in heme and chlorophyll biosynthesis, and its substrates are the targets of salt toxicity leading to massive oxidative stress. The increment of protoporphyrinogen IX oxidase would contribute to alleviate oxidative stress in salt-stressed maize chloroplasts. Monogalactosyl diacylglycerol synthase and calcium-sensing receptor were salt-reduced in the maize chloroplasts. These two enzymes are involved in membrane maintenance and Na^+^ sensing, respectively. These results provide valuable informationfor future studies on the molecular mechanisms of salt tolerance in maize chloroplasts.

High/low temperature and light effects on maize photosynthetic apparatus have been investigated (Caffarri et al., 2005). Low temperature led to decreases of chlorophyll contents and *F*_v_/*F*_m_ in maize indicating that low temperature could cause photoinhibitory damage to the PSII reaction center. In addition, maize plants grown under low light and high temperature conditions exhibited an increased value of non-photochemical quenching. Under multiple temperature and light conditions, low temperature is the principal factor that affects protein expression in maize thylakoid membranes. For instance, LHCII contents in maize plants under low temperature were higher than under high temperature. Minor antenna proteins were decreased compared to the LHCII proteins in maize plants grown under low temperature (**Figure 1J**). In addition, nine LHCb1, two LHCb2, and three LHCb3 protein spots were positively detected by corresponding antibodies on 2DE gels (Caffarri et al., 2005; **Figure 1J**). They presented diverse expression patterns under different temperature/light conditions. This suggests that different genes were translated into proteins of thylakoid membranes in response to environmental stress, which might be a basic mechanism in the C_4_ photosynthetic apparatus for environmental adaptation.

## CONCLUSION

The maize chloroplast is a good model for studying the C_4_ photosynthetic mechanism. The development of large-scale quantitative proteomics approaches together with the availability of maize genome sequences has provided a high-throughput platform with high resolution and sensitivity for analyzing protein expression patterns in the M and BS chloroplasts of maize. The quantitative proteomics information acquired to date provides new insights into the specific C_4_ chloroplast biogenesis, M and BS differentiation, and stress response. However, the photosynthetic machinery and metabolic mechanisms are too complicated to be interpreted by just using quantitative protein profiles. Specialized protein complexes, protein-protein interaction, and post-translational modifications have been proposed to play key roles in photosynthesis. Thus, further proteomics studies should focus on the analysis of large-scale protein modifications and interactions to enhance our understanding of the protein networks in C_4_ photosynthesis.

## Conflict of Interest Statement

The authors declare that the research was conducted in the absence of any commercial or financial relationships that could be construed as a potential conflict of interest.
